# Experimental demonstration of passive microwave pulse amplification via temporal Talbot effect

**DOI:** 10.1038/s41598-023-42361-1

**Published:** 2023-09-15

**Authors:** Vinicius M. Pepino, Achiles F. da Mota, Ben-Hur V. Borges

**Affiliations:** 1 Department of Electrical and Computing Engineering, University of São Paulo, Campus of São Carlos, São Carlos, SP CEP 13566-590 Brazil; 2 Department of Electrical Engineering, University of Brasília, Brasília, DF CEP 70910-900 Brazil

**Keywords:** Electrical and electronic engineering, Characterization and analytical techniques

## Abstract

The temporal Talbot effect is a passive phenomenon that occurs when a periodic signal propagates through a dispersive medium with a quadratic phase response that modulates the output pulse repetition rate based on the input period. As previously proposed, this effect enables innovative applications such as passive amplification. However, its observation in the microwave regime has been impractical due to the requirement for controlled propagation through a highly dispersive waveguide. To overcome this challenge, we employed an ultra-wide band linearly chirped Bragg grating within a standard microwave X-Band waveguide. By utilizing backwards Talbot array illuminators aided by particle swarm optimization, we achieved passive amplification with a gain of 3.45 dB and 4.03 dB for gaussian and raised cosine pulses, respectively. Furthermore, we numerically verified that with higher quality substrates this gain can be theoretically increased to over 8 dB. Our work paves the way for numerous applications of the Talbot effect in the microwave regime, such as temporal cloaking, sub-noise microwave signal detection, microwave pulse shaping, and microwave noise reduction.

## Introduction

The Talbot effect, first observed in 1836, is a well-known phenomenon in wave optics where a periodic structure is self-replicated at regular intervals when a coherent beam of light passes through it. In the Talbot effect, the replicated structure appears at a distance equal to the original grating's length, known as the Talbot distance^1,2^. The temporal Talbot effect (TTE), in turn, is a similar phenomenon in the time domain, first proposed in 1981^3^ and experimentally verified in 1993^4^. It occurs when a pulsed light source, such as a pulsed laser, is sent through a first order dispersive medium. The replicated pulses, known as the Talbot fractional images, are observed at regular intervals, which are shorter than the period of the input signal^5–10^, and can even convert continuous wave (CW) signals into pulses by propagating the input signal through a first-order dispersive medium^11^. The temporal Talbot effect has found numerous applications in signal processing, including pulse rate multiplication^9,12–14^, pulse generation^11,15^, passive amplification^16,17^, signal denoising^18^, sub-noise detection^19^, pulse compression^20^, and temporal cloaking^21,22^.

Despite its potential, every TTE implementation to date has been achieved with optical fibers because it requires long propagation lengths, high dispersion, and low loss. For instance, previous TTE implementations have utilized either single-mode standard fibers^4,16^, requiring up to kilometers of fibers, or linearly chirped fiber Bragg gratings^23,24^, which requires only a few centimeters^25^. Dispersion compensating fibers, such as Bragg^26,27^ or photonic crystal fibers^28–30^, have also been used for this purpose^9,17^. The high dispersion coefficient of these fibers originates from a rapidly varying electric field profile as the operating wavelength is changed, leading to a fast change in the effective index. However, these fibers have limited dispersive bandwidth and their cross-sectional area is large compared to the wavelength, which is problematic for microwave frequencies. In contrast, fiber Bragg gratings can be realized in single mode waveguides, significantly reducing the cross-sectional area, close to the diffraction limit, which is crucial for microwave applications. Furthermore, the dispersive bandwidth of fiber Bragg gratings depends only on the initial and final period of the grating, making them a more versatile option for implementing TTE in the microwave and mm-wave range. More recently, we have theoretically proposed passive amplification in the terahertz range using a low-loss all-silicon rectangular waveguide with metamaterial clad in a Bragg fiber-type configuration^17^, suggesting that the TTE may find broader use in the future.

Despite the numerous applications of TTE, its implementation in longer wavelengths requires large structures. As a result, only its spatial counterpart has been realized in the microwave range, with a limited number of applications, such as antenna power combining^31,32^. Meanwhile, the previously mentioned myriad of applications of TTE remains unexplored. Additionally, lower repetition rates require either extremely high dispersive media or prohibitively long propagation lengths, with the latter simultaneously complicating the device's fabrication besides increasing propagation losses. Notably, the propagation length necessary for TTE is proportional to the square of the signal period, which is limited by both the carrier frequency and the operational bandwidth of the dispersive waveguide. Usually, there are two approaches to overcome these limitations. Firstly, by increasing the dispersion coefficient of the propagating medium and, secondly, by increasing its dispersive spectral bandwidth. Our work demonstrates that, in cases where there is a trade-off between maximum dispersion and dispersive bandwidth, the latter is preferred. This approach allows for shorter pulse periods and propagation lengths, making the TTE implementation more feasible in the microwave and mm-wave ranges.

In this paper, we present the first experimental demonstration of the TTE in the microwave regime using an ultra-wideband linearly chirped Bragg grating (LCBG) fabricated with stereolithography (SLA) technology. The LCBG is designed to operate over the entire X-band (8.2–12.4 GHz) and is incorporated into a standard WR-90 metallic waveguide. To extract the output signal, we use a broadband microwave circulator due to the reflective nature of the LCBG. By varying the input pulse repetition rate, we observe the complete Talbot carpet in a hybrid numerical-experimental strategy and experimentally observe the Talbot fractional images relative to 1/2 and 1/3 of the Talbot length. Moreover, we utilize the backward Talbot array illuminator (BTAI) technique to generate gaussian and raised cosine pulses with a passive gain of 3.44 dB and 4.03 dB, respectively. The BTAI is a recently developed application of the TTE for continuous wave (CW)-to-pulse conversion. It is based on the Talbot array illuminator^11^ and provides passive amplification, accounting for all non-idealities of the dispersive medium^17^.

This paper is organized as follows. First, we present the theoretical background of the propagation length requirements for the TTE and discuss the trade-offs between the dispersion coefficient and dispersive bandwidth in a LCBG. Then, we detail the design strategy of our LCBG, the fabrication procedure, and experimental frequency response measurements. We then show our simulation and experimental methods and present the simulated and experimental results of the temporal Talbot carpet and BTAI pulse generation. Finally, we summarize our findings and provide some concluding remarks. In Supplementary Information, we present the frequency-dependent complex permittivity of the SLA resin and explore the potential for passive amplification with lower loss dielectrics, which can potentially yield gains over 8 dB.

## Theoretical background

### Temporal Talbot effect limitations and implementation requirements

The TTE occurs when a periodic signal (${U}_{o}\left(t\right)$) propagates through a first-order dispersive media with propagation constant given by $\beta \left(\omega \right)=\left[{\beta }_{0}+{\beta }_{1}\omega +{\beta }_{2}{\omega }^{2}/2\right]$^17^, where *ω* is the angular frequency and ${\beta }_{n}\left({\omega }_{0}\right)={\mathrm{d}}^{\left(\mathrm{n}\right)}\beta /\mathrm{d}{\omega }^{n}{|}_{\omega ={\omega }_{o}}$. The temporal and spatial evolution of the signal ($U\left(t,z\right)$) is calculated using the relation^17^:

1 $$U\left(t,z\right)=\sum_{l=-\infty }^{\infty }{a}_{l}{\mathrm{e}}^{\mathrm{j}\frac{2\pi l\left(t-{\beta }_{1}z\right)t}{T}}{\mathrm{e}}^{-\mathrm{j}\left(\frac{2{\pi }^{2}{l}^{2}{\beta }_{2}}{{T}^{2}}\right)z},$$

where *t* is the time, *z* is propagated distance, ${a}_{l}$ is the *l*th frequency harmonic of ${U}_{o}\left(t\right)$ given by its Fourier series. As explained in^17^, the first exponential term in (1) is responsible for the group delay, while the second term distorts the pulses’ shape by applying a linear chirp. For the specific cases where $z={z}_{TT}/q$, called fractional Talbot lengths, (1) can be rewritten as^17^,

2 $$U\left(t,z=\frac{{z}_{TT}}{q}\right)=\left[{U}_{0}\left(t-{\beta }_{1}\frac{{z}_{TT}}{q}-{e}_{q}\frac{T}{2}\right)\right]*\left[\frac{1}{\sqrt{q}}\sum_{n=0}^{q-1}{\mathrm{e}}^{\mathrm{j}{\zeta }_{n}}\delta \left(t-n\frac{T}{q}\right)\right],$$

3 $${z}_{TT}=\frac{{T}^{2}}{2\pi \left|{\beta }_{2}\right|},$$

where *q* is an integer, ${e}_{q}=1$ or 0 whenever $q$ is even or odd, the second term of the convolution is a pulse train with period *T*/*q* and constant phase ${\zeta }_{n}={\zeta }_{0}+{e}_{q}\pi +\left(s/q\right)\pi {n}^{2}$ with period *T*/*q*, while the first term corresponds to the input signal ${U}_{0}$ shifted in time. In summary, after the pulse propagates a distance $z={z}_{TT}/q$, $U(t,z)$ will consist of *q* repetitions of ${U}_{0}$ scaled by $1/\sqrt{q}$.

This formalism can also be employed to obtain the Talbot carpet, a fractal pattern of sub-images with an ever-increasing repetition rate that results from the Talbot effect. The Talbot carpet consists of observing $U(t,z)$ as it propagates through a distance ${z}_{TT}/q$ and observing the *q* repetitions of *U*_*0*_. Although it is the most common approach to generate the Talbot carpet, in applications with fixed propagation length $\left(L={z}_{TT}/q\right)$, the carpet can also be generated by varying $T$ instead of *z*. In this sense, only specific values of *T* fulfill the requirements for the TTE fractional images, and, by applying the definition of *L* in (3) the *q* repetition rate occurs when

4 $$T=\sqrt{2\pi q\left|{\beta }_{2}\right|\mathrm{L }.}$$

However, to generate the Talbot carpet, the medium must have a first-order dispersion profile over the bandwidth *B* occupied by the signal *U*_*0*_. Assuming *U*_*0*_ occupies a fraction of the period *KT* (where $0<K<1$), the signal occupies a bandwidth $B=1/KT$. Moreover, defining ${\phi }_{2}=\left|{\beta }_{2}L\right|$ as the second derivative of the accumulated phase over the propagation length, (4) can be rewritten as

5 $$2{\pi \phi }_{2}=\frac{q}{{K}^{2}{B}^{2}}.$$

A key parameter in dispersive structures is its total available dispersive bandwidth. In the microwave regime, achieving high β_2_ over a large bandwidth is a challenging task. Consequently, the Talbot carpet has never been realized in this frequency range. However, increasing *B* (or reducing *T*) quadratically decreases the necessity of large β_2_, as seen in (5). By recasting (3) as a dependance of the signal bandwidth, we can compare it with the dispersion coefficient-dispersive bandwidth relationship of dispersive structures such as a LCBG, which is thoroughly analyzed in the next Subsection. This comparison allows us to determine the optimal parameters of the LCBG. In other words, it helps us decide if we should use a wideband structure to take advantage of lower values of *T* and therefore reduce the necessary ${\phi }_{2}$, or if we should sacrifice the dispersive bandwidth to increase ${\phi }_{2}$ of the LCBG.

### The linearly chirped Bragg grating characteristics

The LCBG is a Bragg grating structure in which the grating period $\Lambda$ varies linearly along its length^23^. Each period is composed of two quarter wavelength sections with different refractive indexes, *n*_*1*_ and *n*_*2*_, resulting in a broad reflective spectrum with a varying group delay $\tau (\omega )$. Quarter wavelength plates are reflective structures, and by linearly increasing $\Lambda$, the initial plates reflect longer wavelengths while the final ones reflect shorter wavelengths. As a result, longer wavelengths experience a shorter round-trip time, and the opposite occurs for shorter wavelengths. To further understand the impact of this device on the Talbot effect, let us consider a LCBG with *N* periods, where the *i*-th period (${\Lambda }_{i}={\lambda }_{0}^{i}/4{n}_{1}+{\lambda }_{0}^{i}/4{n}_{2}$) is responsible for reflecting the frequency *f*_*i*_ (with wavelength ${\lambda }_{0}^{i}=c/{f}_{i}$). The delay experienced by *f*_*i*_ (${\tau }_{i}$) is given by,

6 $${\tau }_{i}=\frac{2}{{V}_{g}}\sum_{j=1}^{i}{\Lambda }_{j},$$

where the summation represents the path traveled by the wave (multiplied by two since it is a round trip) and ${V}_{g}$ is the group velocity, assumed here as not dispersive since the filling factor of all periods is the same, and the materials are essentially non-dispersive in the frequency range of interest. To adequately perform TTE, the device should have first-order dispersive behavior and $2\pi {\phi }_{2}=\partial \tau /\partial f$ must be constant, thus $\Delta {\tau }_{i}/\Delta {f}_{i}=\mathrm{cte}$. To ensure this behavior,

7 $$2\pi {\phi }_{2}=\frac{\Delta {\tau }_{i}}{\Delta {f}_{i}}=\frac{2}{c/{V}_{g}}\left(\frac{{n}_{1}+{n}_{2}}{{n}_{1}{n}_{2}}\right)\left(\frac{1}{\left({f}_{i+1}-{f}_{i}\right){f}_{i+1}}\right),$$

where ${V}_{g}/c={n}_{eff}^{\mathrm{LCBG}}$ is the effective refractive index of the LCBG.

Equation (7) provides a roadmap on how to design a LCBG. Once the device’s initial frequency (${f}_{0}={f}_{ini}$) and desired ${\phi }_{2}$ are known, the dispersive behavior is achieved by calculating the *N* steps of ${f}_{i+1}$ using (7). Moreover, it is interesting to note that even if the procedure starts by defining the device’s final frequency (${f}_{N}={f}_{fin}$) and calculating ${f}_{i-1}$**,** the obtained ${\Lambda }_{i}$ would remain the same. In practical terms, it means that the device would present the same $|{\phi }_{2}|$ with opposite signs when excited from either side. It is crucial to note that ${\Lambda }_{i}$ is proportional to ${\lambda }_{0}^{i}$, and therefore, using small frequency steps (for a continuous reflective profile) would result in a lengthy structure. However, for the purpose of qualitatively understanding the relationship between the dispersion coefficient and the dispersive bandwidth of a LCBG, this idealization is justified. After defining ${\Lambda }_{i}$, ${\phi }_{2}$ can be calculated as,

8 $$2\pi {\phi }_{2}=\frac{2{L}_{0}}{\sum \Delta {f}_{i}}{n}_{eff}^{\mathrm{LCBG}},$$

where *L*_*0*_ is the total length of the device. A complete rigorous analysis of the LCBG can be found in^23^. For a fixed grating length and refractive index (which are determined by the materials used), the dispersion coefficient is inversely proportional to the dispersive bandwidth, ${\phi }_{2}\propto 1/\sum \Delta {f}_{i}$. Nevertheless, according to (5), the required ${\phi }_{2}\propto 1/{B}^{2}$ and $\sum \Delta {f}_{i}\ge B$. Thus, maximizing $\sum \Delta {f}_{i}$ while reducing ${\phi }_{2}$ is desired for implementing the TTE.

So far, the LCBG formalism has been developed for a wave propagating in free space. However, to facilitate the measurement procedure, we place the LCBG inside a WR90 waveguide. In this sense, the *i*-th guided wavelength ${\lambda }_{o}^{i}$ inside a rectangular metallic waveguide operating in the TE_10_ mode is given by:

9 $${\lambda }_{g}^{i}=\frac{{\lambda }_{o}^{i} }{\sqrt{1-{\left(\frac{{\lambda }_{o}^{i}}{2W}\right)}^{2}}},$$

where ${\lambda }_{o}$ is the free space wavelength, and *W* is the waveguide cross-section width. Note that the relation between ${\lambda }_{0}^{i}$ and ${f}_{i}$ is not inversely proportional, and *V*_*g*_ becomes dispersive when the LCBG is placed inside a waveguide. In this case, to maintain the relation $\Delta {\tau }_{i}/\Delta {f}_{i}=\mathrm{cte}$, we have

10 $$2\pi {\phi }_{2}=2{n}_{eff}^{guide}\left(f\right)\left(\frac{{n}_{1}+{n}_{2}}{{n}_{1}{n}_{2}}\right)\left(\frac{1}{\left({f}_{i+1}-{f}_{i}\right){f}_{i+1}}\right),$$

11 $${n}_{eff}^{guide}\left(f\right)=\sqrt{1-{\left(\frac{{\lambda }_{o}^{i}}{2W}\right)}^{2}}{n}_{eff}^{\mathrm{LCBG}}.$$

Using the same procedure to design the LCBG in free space, we first define the initial frequency (${f}_{0}={f}_{ini}$) and the desired ${\phi }_{2}$. Then, we calculate ${f}_{i+1}$ and ${\Lambda }_{i+1}$, until the *N*-th period. Finally, ${\phi }_{2}$ can be obtained as,

12 $$2\pi {\phi }_{2}=\frac{2{L}_{o}}{\mathrm{c\Delta }f}\left(\frac{{n}_{1}+{n}_{2}}{{n}_{1}{n}_{2}}\right)\sqrt{1-{\left(\frac{{\lambda }_{o}^{N}}{2W}\right)}^{2}},$$

It is worth noting that the dispersive behavior of ${n}_{eff}^{guide}$ results in the structure exhibiting different ${\phi }_{2}$ values when excited from different sides. In addition, the *i*-th period also changes when the structure is designed from the initial or final frequency. Therefore, we conclude that the LCBG exhibits different dispersive characteristics when excited from either the shorter or longer period ends, as experimentally confirmed in the next section.

## Design, fabrication and characterization

To assess the TTE in the microwave regime, we design the LCBG to operate in the X-band (8.2–12.4 GHz) due to its high central frequency, allowing fast modulation rates (shorter *T*). Another advantage of using the X-band is the possibility of placing the structure inside a standard metallic waveguide WR-90 (rectangular cross-section, dimensions 22.86 × 10.16 mm), facilitating the measurements. The LCBG is built using SLA printing technology, where the resin permittivity $\varepsilon {\prime}$ and loss tangent $\mathrm{tan}\delta$ at 10 GHz are equal to 2.7 and 0.027, respectively. The dispersive behavior and the approach to characterize the resin^33^ are presented in Supplementary Information.

As well known, the building blocks of the Bragg reflector demand two different materials, chosen here as the printed resin spaced by air layers. To guarantee operation inside the desired dispersive bandwidth, we choose the initial and final period corresponding to free space wavelengths of ${\lambda }_{o}^{0}=25$ mm, and ${\lambda }_{o}^{N}=40$ mm, corresponding to the lower and upper limits of the X-Band. From (12), the calculated value of the dispersion coefficient assuming ${L}_{o}=0.3$ m (chosen to fit inside two 6" waveguide sections) is $2\pi {\phi }_{2}=0.47$ ns^2^ when excited from the longer period end (or 0.28 ns^2^ if we chose the shorter period end instead), suggesting that exciting the LCBG from the longer end is preferable. Hereon, we define ports 1 and 2 as the excitation at the shorter and longer period ends, respectively.

**Figure 1:**
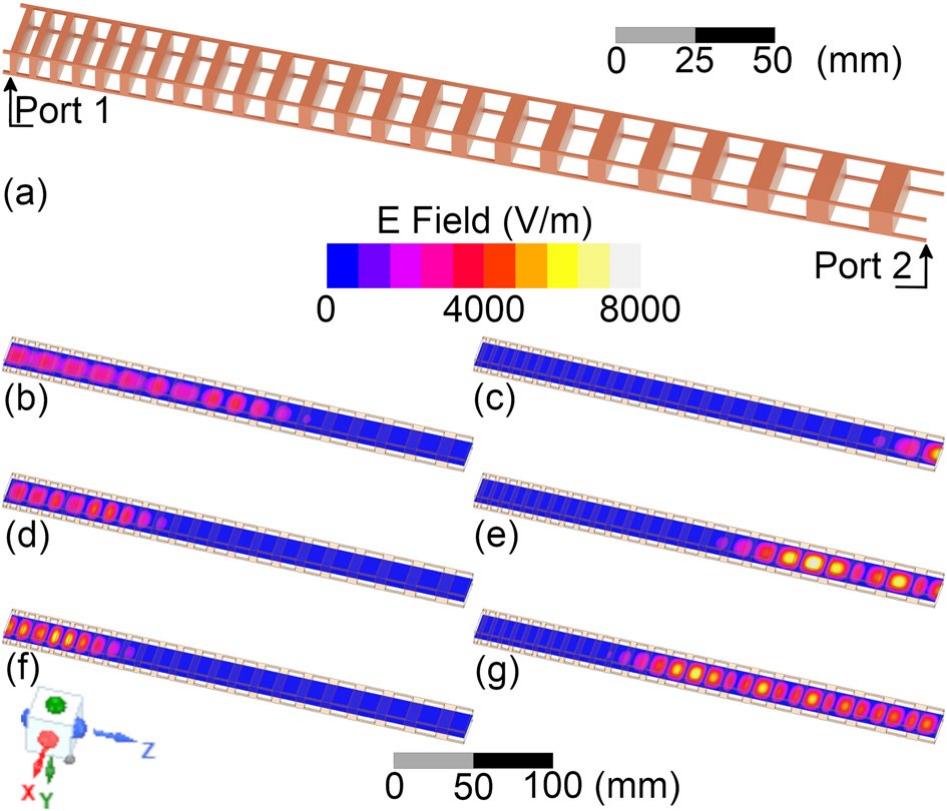
(**a**) Three-dimensional model of the designed LCBG. Electric field at 8 GHz with the excitation at ports (**b**) 1(left side) and (**c**) 2(right side). Electric field at 10.5 GHz with the excitation at ports (**d**) 1 and (**e**) 2. Electric field at 13 GHz with the excitation at port (**f**) 1 and (**g**) 2. Note how the excitation from port 2 (right side figures) result in a shorter penetration distance, which results in lower losses, and a greater difference in the penetration distance from 8 and 13 GHz, which results in a higher dispersion coefficient.

A model of the designed LCBG is presented in Fig. 1a. Figure 1b–g show the simulated electric field inside the waveguide, simulated with Ansys HFSS software, for *f* = 8 GHz in (b) and (c), 10.5 GHz in (d) and (e), and 13 GHz in (f) and (g), when excited from port 1 [(b), (d) and (f)] or port 2 [(c), (e), and (g)]. As expected, the electric field propagates a shorter distance for lower frequencies when excited from port 1 than when excited from port 2. Moreover, longer propagation entails higher attenuation since the resin presents a considerable loss, which can be seen by the weaker electric field when the LCBG is excited from port 1. Another important aspect is that a higher $2\pi {\phi }_{2}$ implies a faster delay variation, meaning that the wave excited from port 2 (higher dispersion) experiences a faster round trip (less lossy) when the frequency is changed. This behavior means the structure presents less loss and more linear reflection when excited from port 2.

**Figure 2:**
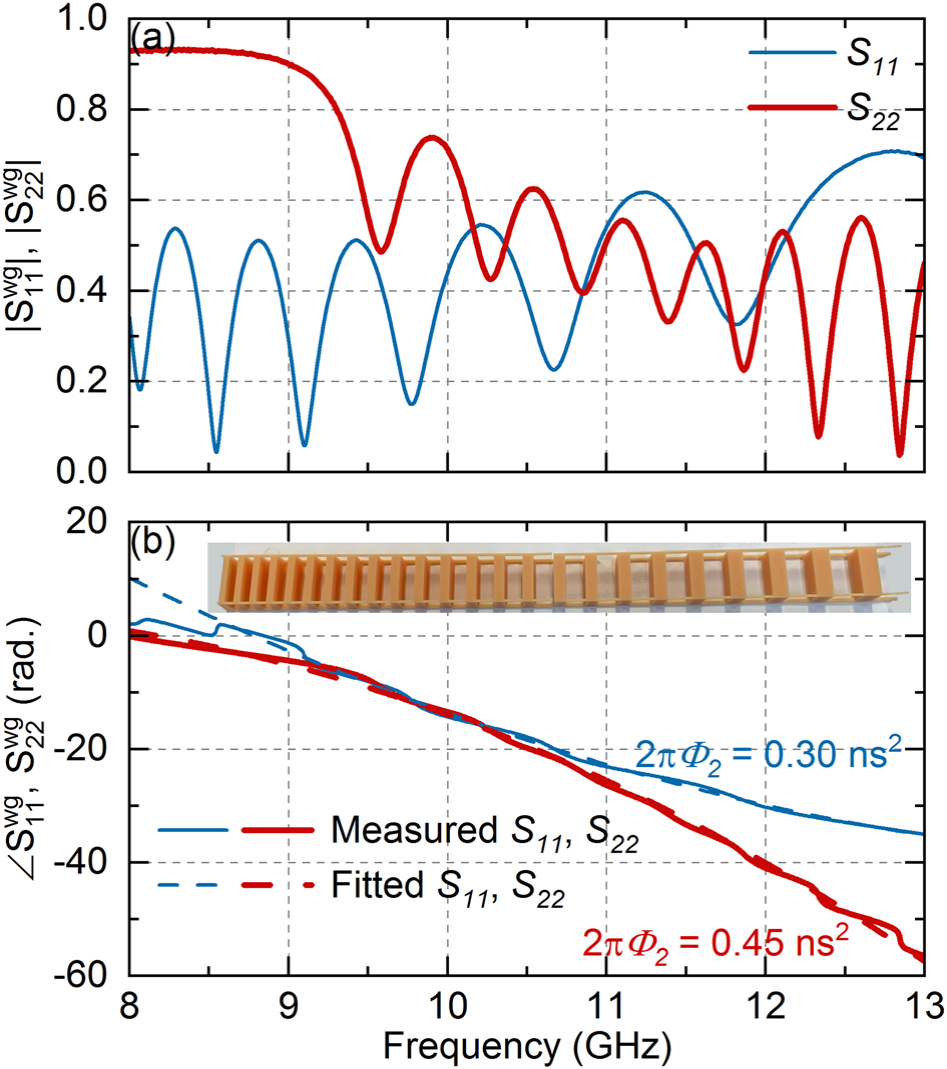
(**a**) Measured values of the reflection coefficients of the fabricated LCBG inside a WR-90 waveguide when excited from port 1 (thin blue lines) and 2 (thick red lines). (**b**) Unwrapped argument of the reflection coefficients (solid line) and its respective quadratic fits (dashed lines). Note that exciting the LCBG from port 2 is preferred, both due to higher reflected average amplitude and higher dispersion coefficient, as well as a broader dispersive bandwidth (look at the phase response below 8.5 GHz). Fabricated LCBG is shown in inset of (**b**).

All these observations are confirmed by the reflection coefficients of the filled waveguide, ${S}_{11}^{WG}$ and ${S}_{22}^{WG}$, measured by a Rohde & Schwartz ZVA-40 Vector Network Analyzer (VNA), shown in Fig. 2. Figure 2a and b show the amplitude and phase, respectively, of ${S}_{\mathrm{11,22}}^{WG}$ in thin blue lines and thick red lines, respectively. Figure 2b shows the fabricated LCBG as an inset. Moreover, we use a second-order polynomial fit to determine the average value of $\left|2\pi {\phi }_{2}\right|$ because the LCBG non-idealities (both due to the low number of periods and high dielectric loss tangent) create oscillations in the ideal quadratic phase response. The polynomial fit is shown as a dashed line in Fig. 2b and provides an average value of $\left|2\pi {\phi }_{2}\right|$ of 0.30(0.45) ns^2^ when excited from port 1(2), supporting the validity of the proposed approach in creating a first-order dispersive medium. It is important to highlight that although the oscillations in the ideal phase response create non-idealities in the TTE, the effect is still achievable, as observed in previous works with non-ideal dispersive media^34^.

## Methods

### Simulation

Ansys HFSS 2022 is used to simulate the frequency response of our LCBG. The relative permittivity and loss tangent of the 3D printing resin used in simulation are equal to 2.7 and 0.027, respectively. To simulate the waveguide walls, finite conductivity boundary conditions are used with conductivity $\sigma =5.8 \times {10}^{7}$ S/m, which is the electric conductivity of copper. To simulate the time waveforms obtained in the LCBG, we use MATLAB R2022a to multiply the Fourier series of the input signal by the frequency response of the LCBG (either simulated or measured with a R&S ZVA40 Vector Network Analyzer).

### Experiments

The LCBG is printed using Anycubic craftsmen resin in a Anycubic Photon Mono X 6 k printer. Layer exposure time is set to 40 s in the first four layers and 1.7 s for the rest. The LCBG is then placed inside Pasternack PE-W90S001-6 microwave waveguides terminated by a Pasternack PE6815 waveguide load. The reflected signal is obtained with a Pasternack PE83CR002 8–18 GHz circulator. A R&S ZVA40 Vector Network Analyzer is used for frequency domain measurements while a Keysight DSOV134A oscilloscope (80 GS/s, 13.6 GHz bandwidth) and a Keysight M8195A arbitrary wave generator (65 GS/s, 25 GHz bandwidth) are used for time domain measurements, such as Talbot carpet visualization and pulse generation.

## Results and discussion

### Talbot carpet

**Figure 3:**
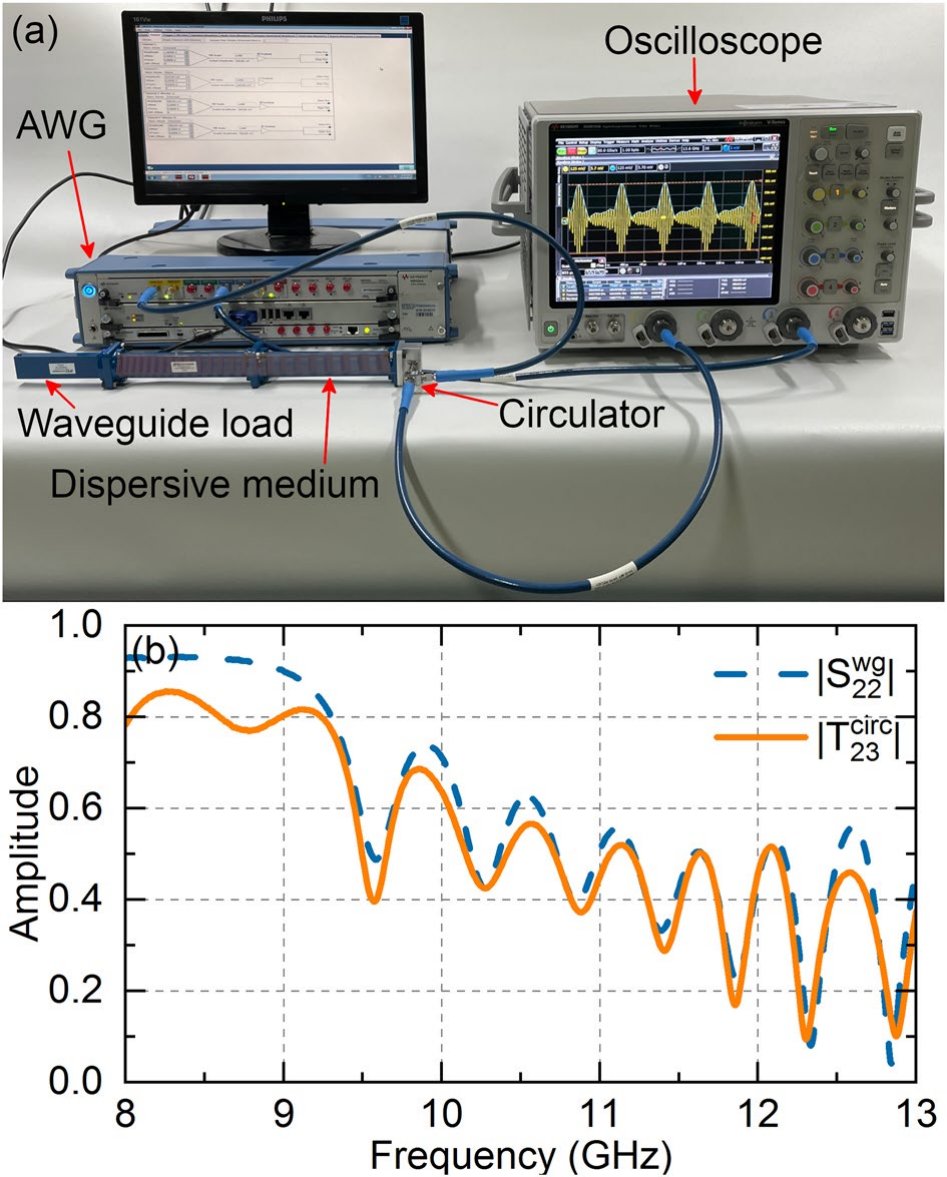
(**a**) Experimental setup for measuring the TTE. The two waveguide sections are loaded with our LCBG (dispersive medium shown in transparency to visualize its full length inside the waveguide), with a broadband waveguide load in port 1 and a microwave circulator in port 2. Ports 2 and 3 of the circulator are connected to a oscilloscope and a AWG respectively. (**b**) Effect of the circulator on the frequency response. ${T}_{23}^{circ}$, shown in solid orange lines, adds extra losses to the system when compared to ${S}_{22}^{wg}$, in dashed blue lines. To perform a correct design optimization, we use ${T}_{23}^{circ}$ in all of our simulations. The phase response does not change and thus is omitted.

The experimental setup for implementing the TTE is illustrated in Fig. 3a, where the LCBG is inserted inside two straight sections of WR-90 waveguide, terminated with a matched waveguide load. To measure the reflected signal, we use a broadband circulator (8–18 GHz) with its port C1 connected to port 2 of the waveguide. Port C2 of the circulator is connected to a Keysight DSOV134A 80 GS/s oscilloscope (with a 13.6 GHz bandwidth), while port C3 is connected to a Keysight M8195A 64 GS/s arbitrary wave generator (AWG) with a 25 GHz bandwidth. To account for the non-flat frequency response of the circulator over the entire bandwidth, we characterize the transmission from port C3 to port C2 of the circulator with port 1 loaded with the dispersive waveguide. The resulting transmission coefficient ${T}_{23}^{circ}$ is shown in Fig. 3b and compared with ${S}_{22}^{WG}$, where the former exhibits a slightly lower amplitude than the latter due to the loss inserted by the circulator. Importantly, the phase response and the dispersion coefficient are not significantly affected.

To visualize the Talbot carpet in a fixed propagation length, we must vary the input period *T*. From (4), $T=\sqrt{2q\pi \left|{\beta }_{2}\right|L}$, and thus we can correlate the propagation length with the input period, resulting in periods of 0.95 ns, 1.16 ns, and 1.34 ns for *q* = 2, 3, and 4, respectively. Due to phase oscillations and other non-idealities in the dispersive profile inducing higher order dispersion coefficients^34^, we expect the necessary period to be higher than the calculated value for an ideal structure. Thus, we sweep *T* from 1.0 to 2.0 ns to visualize the temporal Talbot carpet in a hybrid simulation-experimental strategy, where the pulse propagations are numerically calculated by multiplying the Fourier series spectrum of the input signal by the measured frequency response ${T}_{23}^{circ}$ shown in Fig. 3b, then we reconstruct the signal in the time domain. We use Gaussian input pulses defined as:

13 $${U}_{o}\left(t\right)={\mathrm{e}}^{-\left(\frac{{t}^{2}}{2{T}_{o}^{2}}\right)},$$

14 $${T}_{o}=\frac{T{K}_{FWHM}}{2\mathrm{ln}\surd 2},$$

where *K*_*FWHM*_ is the relationship between the input period and the pulses' full width at half maximum (FWHM), chosen as 10% of the temporal slot *T* to avoid pulse overlapping when the repetition rate is multiplied by the TTE. The carrier frequency is chosen to be 10.5 GHz, and we limit the bandwidth to 8–13 GHz, which is below the cutoff of the second mode of the WR-90 waveguide. The normalized output intensity is mapped in terms of the period *T*, which indirectly represents the propagation length and the normalized time *t*/*T*.

**Figure 4:**
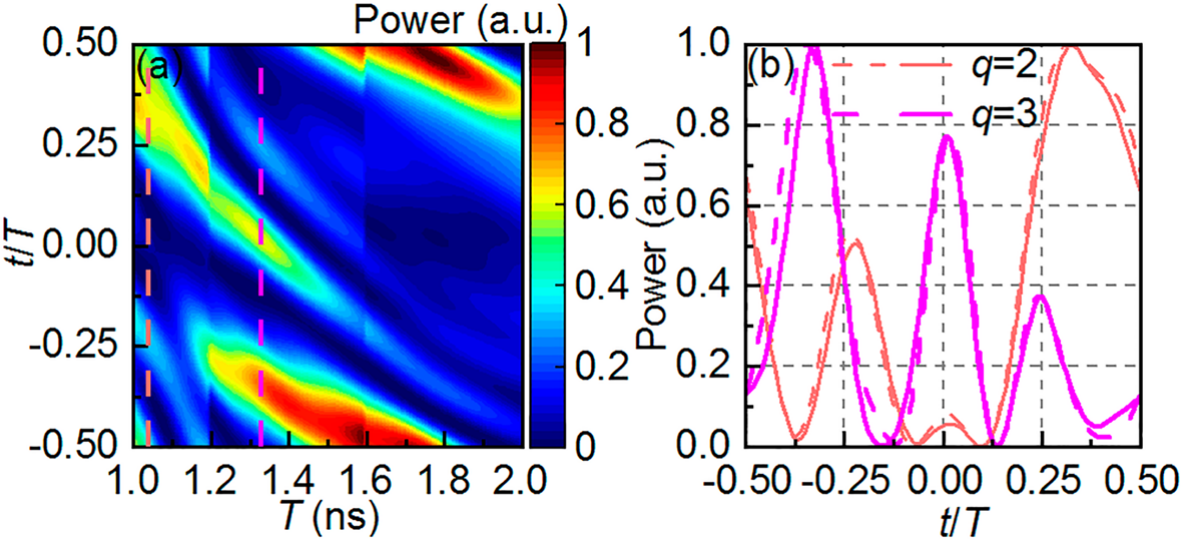
(**a**) Talbot carpet obtained via a hybrid numerical/experimental strategy for simulating pulse propagation using the measured frequency response of the LCBG ${T}_{23}^{circ}$. The time axis is normalized by the period *T* and the period axis indirectly represents the propagation length. Talbot fractional images relative to *q* = 2, 3 occur at *T* = 1.04 ns, 1.33 ns, highlighted by red and magenta dashed lines, respectively. Note how the pulse peaks get closer as *T* increases, suggesting the occurrence of other fractional images (*q* > 3) that do not appear properly due to non-idealities in the dispersive profile and limited bandwidth. Discontinuities at *T* = 1.2, 1.6 ns are related to frequency components of the input pulse being outside the dispersive band and thus are filtered out. (**b**) Slices of the highlighted regions in (**a**) (dashed lines) and fully experimental (solid lines) Talbot fractional images for *q* = 2 (red lines) and 3 (magenta lines). Note that, although the pulses shapes and amplitudes are distorted, their periods are divided by 2 and 3, respectively, as expected in Talbot fractional images.

Results are shown in Fig. 4a. The time axis is normalized by the period *T* with the period axis indirectly representing the propagation length. We observe that the results differ from the ideal theoretical Talbot carpet due to the non-idealities in the frequency response, including valleys in the reflection amplitude caused by dielectric losses, oscillations in the phase behavior, and limited bandwidth. The first two cause the system to deviate from the TTE ideal formulation, while the limited bandwidth filters the spectra of the ideal Gaussian pulses and creates secondary peaks, turning the gaussian pulses into sinc-like shapes and broadening its temporal width; this causes partial overlapping of the output pulses. Nonetheless, we observe that the pulse repetition rate changes, typical of the TTE approach. At *T* = 1.04 ns, the resulting pulse period is approximately half of the input period, which corresponds to the fractional Talbot image relative to *q* = 2; at *T* = 1.33 ns, the output signal exhibits a period approximately equal to a third of *T*, or the factional Talbot image relative to *q* = 3, both highlighted in light red and pink dashed lines, respectively. The discontinuities in the simulated carpet at $T=1.2$ ns and $T=1.6$ ns are caused by limited bandwidth filtering extra harmonics of the input signal, resulting in changes in the pulses' shape and amplitude. The difference between the expected pulse periods for *q* equal to 2 and 3, and the obtained values, are caused mainly by the oscillations in the phase response, which add higher order dispersive coefficients to the system^34^. Additionally, it should be noted that as T increases, the output peaks have a shorter period, which is typical of higher *q* values. However, due to the dispersive non-idealities and the heavy pulse overlapping, the signal is heavily distorted, and no extra pulses are observed for $q>3$. The Talbot fractional images for *q* = 2, 3 are plotted in Fig. 4b, where dashed lines refer to slices of Fig. 4a and solid lines refer to fully experimental results. Thin red lines are used for *q* = 2 and thick magenta lines for *q* = 3. Note that experimental results agree very well with simulations.

### Pulse generation via backwards Talbot array illuminators

A novel method of converting CW to pulses using the TTE, the BTAI, was recently theoretically proposed^17^. One advantage of this method is the use of a numerical backpropagation, considering the actual dispersive characteristics of the propagating medium and accounting for all the non-idealities, to optimize the input phase modulation profile that generates the desired pulse shape at the output. Note that from energy conservation, the pulse peak should indeed be higher than the CW amplitude, resulting in a passive gain.

The method works as follows: First, we specify the target output pulse shape. Subsequently, we backpropagate the signal through the dispersive LCBG using the inverse of $[{T}_{23}^{circ}]$. Since the LCBG has a fixed length, there is no need to sweep the propagation length, as suggested in^17^, where *z* is swept to obtain the lowest signal ripple. We then perform phase demodulation on the backpropagated signal to obtain the phase modulation profile, which is used to modulate a continuous wave (CW) signal. Finally, the phase-modulated CW signal is transmitted through the LCBG to generate the desired output pulse train. To optimize the target pulse parameters, such as the period, carrier frequency, and temporal width, we employ the particle swarm optimization (PSO) algorithm to maximize the desired objective, such as the gain *G* (defined as the peak output power divided by the average input power) or signal-to-noise ratio (SNR), defined here as:

15 $$SNR=\frac{{\left[{V}_{output}\left(t\right)\right]}_{RMS}}{{\left[{V}_{output}\left(t\right)-{V}_{target}\left(t\right)\right]}_{RMS}},$$

where *V*_*output*_(*t*) is the output signal and *V*_*target*_(*t*) is the target pulse shape. The *SNR* is used to quantify the distortion in the desired pulse shape that comes from non-idealities in the dispersive profile and amplitude response of the LCBG.

In this study, we investigate the generation of Gaussian and raised cosine (RC) pulses. As previously explained, the narrower the pulse's temporal width, the higher the achievable *G* due to energy conservation. However, generating narrower pulses come at the cost of broadening the spectral bandwidth, which is limited to the X-Band frequency range (8.2–12.4 GHz). Filtering out parts of the spectrum can distort the signal, making it difficult to preserve the shape of a narrow Gaussian pulse. To address this issue, we also generate RC pulses and compare the results with those of Gaussian pulses. These pulses are known for their efficient use of the available bandwidth and reduced inter-symbol interference in communications^35^, and are generated with the use of a RC filter ${H}_{RC}\left(f\right)$, defined as:

16 $${H}_{RC}\left(f\right)=\left\{\begin{array}{ll}1& , \left|f\right|\le \frac{1-{\alpha }_{RC}}{2{T}_{o}^{RC}}\\ \frac{1}{2}\left\{1+\mathrm{cos}\left[\frac{\pi {T}_{o}^{RC}}{{\alpha }_{RC}}\left(\left|f\right|-\frac{1-{\alpha }_{RC}}{2{T}_{o}^{RC}}\right)\right]\right\}& , \frac{1-{\alpha }_{RC}}{2{T}_{o}^{RC}}<\left|f\right|\le \frac{1+{\alpha }_{RC}}{2{T}_{o}^{RC}}\\ 0& , \left|f\right|>\frac{1+{\alpha }_{RC}}{2{T}_{o}^{RC}} \end{array}\right.,$$

where ${T}_{o}^{RC}$ is the temporal distance between the main pulse peak and the first zero crossing, also known as symbol-period, and ${\alpha }_{RC}$ is the roll-off factor. Note that when ${\alpha }_{RC}$ is equal to 0, ${H}_{RC}\left(f\right)$ reduces to an ideal brick-wall filter, and the resulting pulse is a sinc function.

The lower and upper bound constraints for PSO are chosen as ${f}_{c}\in (10.0, 10.6)$ GHz, $T\in (1.0, 2.0)$ ns, and ${K}_{FWHM}\in (0.10, 0.25)$ for gaussian, and ${f}_{c}\in (10.0, 10.6)$ GHz, $T\in (1.0, 2.0)$ ns, ${T}_{o}^{RC}\in (0.2, 0.8)$ ns, and ${\alpha }_{RC}\in (0.00, 0.75)$ for RC pulses. We use PSO to maximize both *G* or *SNR* (while keeping $G>0$ dB) in gaussian pulses, and to maximize *G* in RC pulses. A final optimization is done by maximizing *G* while keeping side peak intensity lower than 1/6 of the main peak intensity. This optimization is found to improve the *SNR* at a small cost in gain. The optimized input parameters are listed in Table 1 for *G* optimized Gaussian (case 1), *SNR* optimized Gaussian (case 2), gain optimized RC (case 3), and gain/side peak optimized RC pulses (case 4). Table 2 summarizes the obtained *G* and *SNR* for each case, both simulated and measured values. Note that the *SNR* optimization provides lower *G* when compared to the gain optimization*,* because we do not set a target gain when optimizing SNR, which may limit its practical application. However, intermediate values of *G* and *SNR* can be obtained by using a weighted objective function in the PSO stage to fit the desired application, such as an intermediate pulse gain with reduced distortion.

**Table 1:** Gaussian and RC BTAI input parameters.

Parameter	Case 1	Case 2	Case 3	Case 4
*f*_*c*_ (GHz)	10.00	10.47	10.20	10.10
*T* (ns)	1.91	1.25	2.00	2.00
*K*_*FWHM*_	0.15	0.17	–	–
${T}_{o}^{RC}$(ns)	–	–	0.32	0.29
${\alpha }_{RC}$	–	–	0.37	0.15

**Table 2:** Gaussian and RC BTAI output parameters.

Parameter		Case 1	Case 2	Case 3	Case 4
G (dB)	Simulated	3.02	0.02	4.20	3.17
	Measured	3.45	1.68	4.03	3.60
SNR (dB)	Simulated	9.29	14.66	11.25	11.52
	Measured	8.89	12.34	7.10	12.12

**Figure 5:**
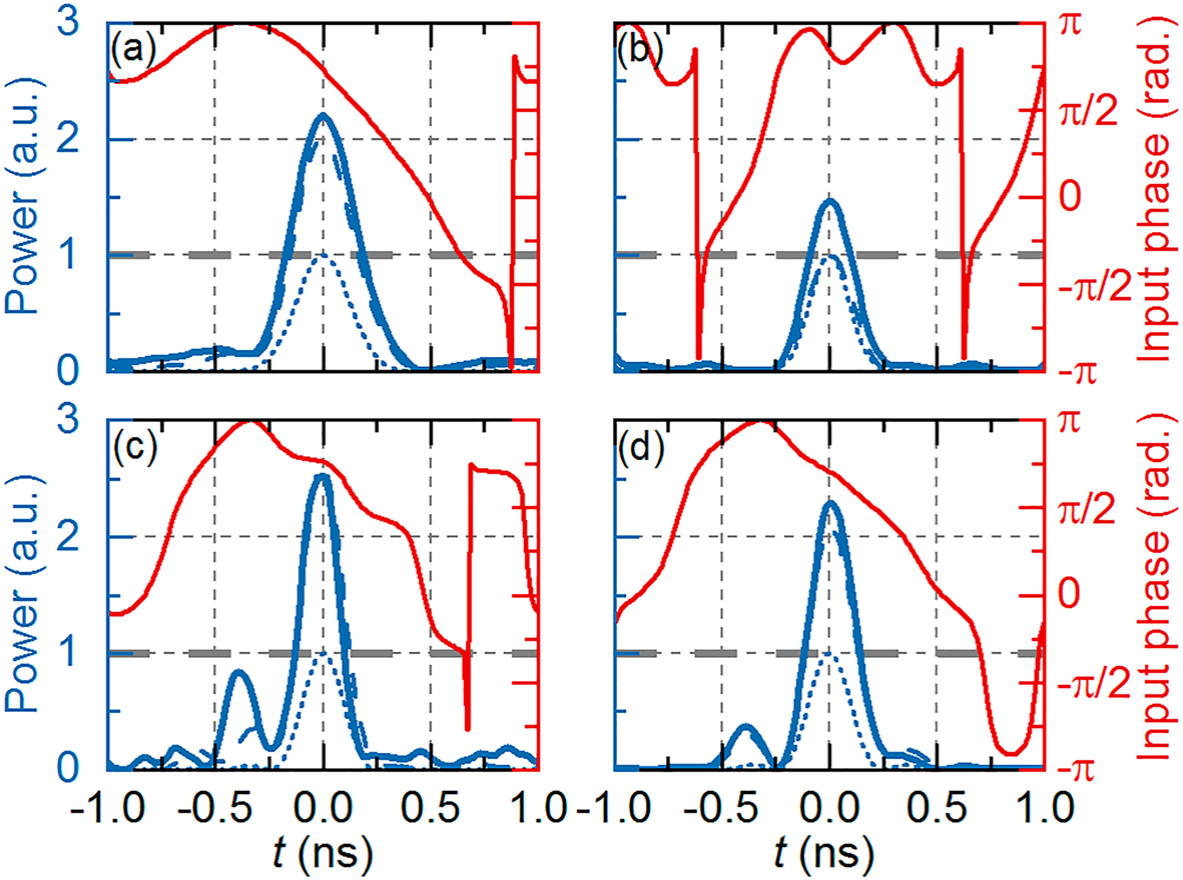
BTAI gaussian-time waveforms for (**a**) gain optimization and (**b**) SNR optimization. RC BTAI time waveforms for (**c**) gain optimization and (**d**) gain optimization with constrained side peak amplitude. The target waveforms are shown in dotted blue lines, with simulated and measured results depicted in dashed and solid blue lines, respectively. Note that the target waveform is plotted with peak intensity equal to 1 to highlight the TTE passive amplification. Optimized input phase profiles are shown in red lines while input signal intensity is shown as a thick dashed horizontal line with 1 a.u. power. Note that the RC pulses achieve higher G than Gaussian while maintaining a good SNR in the side peak limited case.

Figure 5 shows the target (dotted blue lines), simulated (dashed blue lines), and measured (solid blue lines), as well as the input phase profile (solid red lines). Cases 1–4 are shown in (a)-(d). Note that in all cases the input signal is a phase modulated CW with constant amplitude equal to 1, marked by gray dashed lines in all plots. The target signals are shown with amplitude equal to 1 a.u., while the simulated and measured outputs are normalized to the input signals to highlight the passive amplification provided by BTAI. Note that target waveforms represent only the target pulse shape. We do not define a target gain, as the PSO routine is responsible for maximizing it. We observe that RC pulses achieve higher gains than Gaussian ones, while also achieving high *SNR*, confirming the advantage of their flatter frequency spectrum. Case 4 slightly lowers *G*, but it helps reducing the experimental *SNR,* as seen in Fig. 5d, and listed in Table 2.

**Figure 6:**
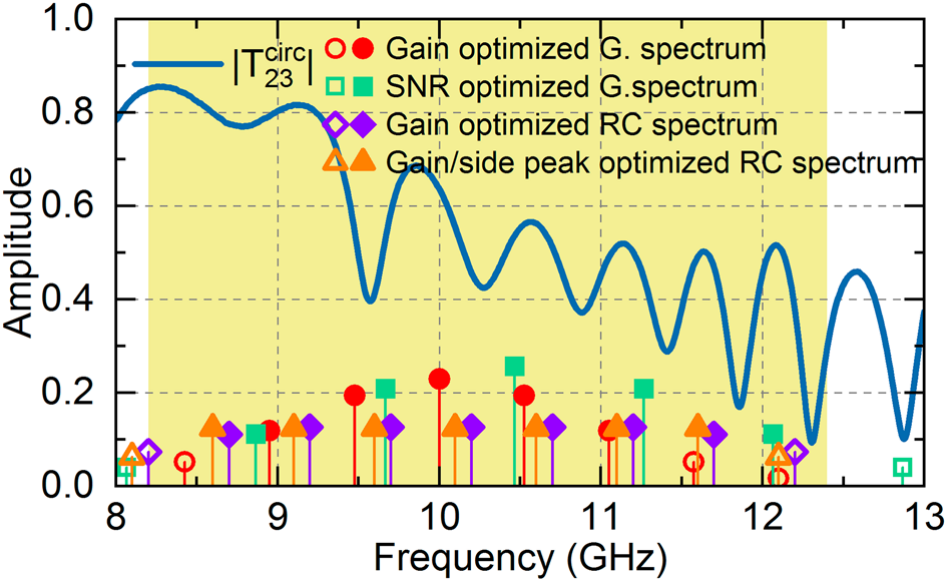
Frequency components of the optimized Gaussian and RC target pulses. The yellow region limits the X-Band. Filled symbols refer to the frequency harmonics responsible for 90% of the pulse’s energy while hollow symbols are the harmonics with less than 10% of the total energy. RC pulses have a more well distributed spectrum which result is a better usage of the available bandwidth, resulting in a narrower pulse width and thus a higher gain. Moreover, the carrier frequency is changed to avoid valleys of ${T}_{23}^{circ}$ (in solid blue lines).

Finally, to better visualize the bandwidth usage efficiency, we show the Fourier spectra of the four pulse trains together with $\left|{T}_{23}^{circ}\right|$ (shown in blue line) in Fig. 6. The light-yellow region limits the X-Band. Filled symbols show the harmonics where 95% of the pulse's total energy is contained. Red circles, green squares, purple diamonds, and orange triangles are used for the spectra of *G*-optimized gaussian, *SNR*-optimized gaussian, *G*-optimized RC, and *SNR*-optimized RC, respectively. Note that the *SNR* optimized Gaussian spectrum has its frequency components localized at lower amplitude values, which explains its lower Gain. Note also that the RC spectra are more well-distributed across the X-Band. This broader spectral width results in narrower temporal width and higher *G*. Furthermore, by making *f*_*c*_ a tuning parameter in the PSO algorithm, we avoid ${T}_{23}^{circ}$ valleys, increasing the realized gain. An experimental gain of 4.2 dB is observed for the RC pulse train, while a gain of 3.45 dB is observed for the gaussian pulse, showing the potential of the TTE for microwave passive amplification and signal generation, especially with the aid of the BTAI technique. Although the high dielectric loss tangent (higher than 0.025) limits the realized gain, the use of better dielectrics can improve the system's performance even further, allowing gains above 8 dB for RC pulses and 6.5 dB for Gaussian pulses, as discussed in Supplementary Information.

## Conclusion and perspectives

In this study, we have successfully demonstrated for the first time the implementation of the TTE in the microwave regime using a LCBG designed and fabricated via SLA printing with low permittivity resin. The LCBG was inserted into a 12-inch WR-90 waveguide, which provided high dispersive behavior over the entire X-Band. By varying the input pulse period, we indirectly observed the Talbot carpet for fractional Talbot lengths of 1/2 and 1/3 for the first time in the microwave regime. Additionally, we generated passive amplified pulses using PSO-aided BTAI, achieving gains of 3.45 dB and 4.03 dB for gaussian and raised cosine pulses, respectively, while maintaining high SNR levels of over 12 dB. We observed that the loss tangent of the LCBG's dielectric is the limiting factor of this structure and showed that higher quality dielectrics could increase the gain to over 8 dB while maintaining high SNR levels. We believe that our results could lead to the development of various applications, such as temporal cloaking, sub-noise microwave signal detection, microwave pulse shaping, and microwave noise reduction, among other applications.

### Supplementary Information

Supplementary Information.

## Data Availability

Data underlying the results may be obtained from the corresponding author, Prof. Ben-Hur V. Borges, upon reasonable request.
