# Supplementary material for: Experimental demonstration of passive microwave pulse amplification via temporal Talbot effect
Source: Sci Rep. 2023 Sep 15;13:15330. doi: 10.1038/s41598-023-42361-1 (PMC10504255; doi:10.1038/s41598-023-42361-1)
Supplement: Supplementary file 1 — Supplementary Information. [file 41598_2023_42361_MOESM1_ESM.pdf]

Supplementary information for Experimental Demonstration of Passive Microwave Pulse Amplification via Temporal Talbot Effect

Vinicius M. Pepino[1], Achiles F. da Mota[2], and Ben-Hur V. Borges[1,*]

[1]Department of Electrical and Computing Engineering, University of São Paulo, Campus of São Carlos, SP, CEP 13566-590, Brazil
[2]Department of Electrical Engineering, University of Brasília, DF, CEP 70910-900, Brazil
[*]benhur@sc.usp.br

Role of dielectric loss in realized passive gain

In section 4, we obtained a maximum gain of 4.03 dB. However, looking at $\left|T_{23}^{circ}\right|$ in Fig. 6, it is easy to see that it its far below the unity value limits the realized gain. Since the LCBG is a reflective structure, we know that its reflectivity can only be reduced by two factors: ohmic losses in the waveguide walls and dielectric losses of the SLA resin (please, see Fig. 7 for frequency dependent relative permittivity (blue curve) and loss tangent (red curve) of the resin). Since the waveguide walls are made of copper, which possesses an extremely high electric conductivity $\sigma = 5.8 \times 10^7$ S/m, we proceed to analyze the role of dielectric losses by simulating the designed LCBG with $\tan\delta = 0.005$, 0.010, 0.015, 0.020, and 0.025. The simulated values of $\left|S_{22}^{wg}\right|$ are shown in Fig. 8, along with their measured values (in black lines). Thick blue and red lines, thin green lines, dashed purple lines, and dotted orange lines are used for $\tan\delta$ ranging from 0.005 to 0.025. Note how higher losses reduce the average value of the reflectivity and increase the peak-valley contrast.
To evaluate how much a reduced reflectivity affects *G*, we simulate all four cases analyzed in Section 4 with varying losses. Table S1 summarizes the simulated *G* and *SNR* values for the different values of $\tan\delta$. Cases 1 to 4 refer to gaussian *G* optimization, gaussian *SNR* optimization, RC *G* optimization, and RC *G* optimization with side peak limitation. The results show that an extra 4.13 dB of gain can be obtained by using a low-loss dielectric for the LCBG. Furthermore, a flatter response in the reflectivity translates into a higher *SNR* for cases 1 and 3. In the cases optimized for *SNR* or side peak intensity, the achieved *SNR* is

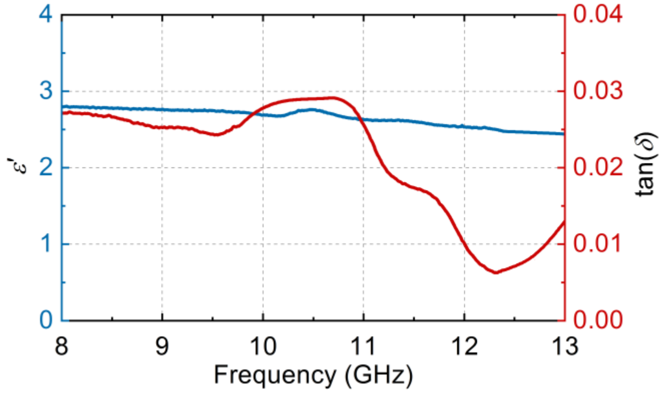

Fig. S1. Measured frequency dependency of the SLA resin used for fabricating the LCBG. Left axis shows the real part of the relative permittivity in blue lines and right side shows the dielectric loss tangent. Note that there is some dispersive behavior to the resin, with its real part reducing from approximately 2.8 to 2.5 as the frequency increases, while the loss tangent experiences a fast decrease in frequencies above 11 GHz.

lower than the measured values. This happens because the phase profile optimization was carried out considering the measured frequency response pattern and thus any change in it will decrease the *SNR*.

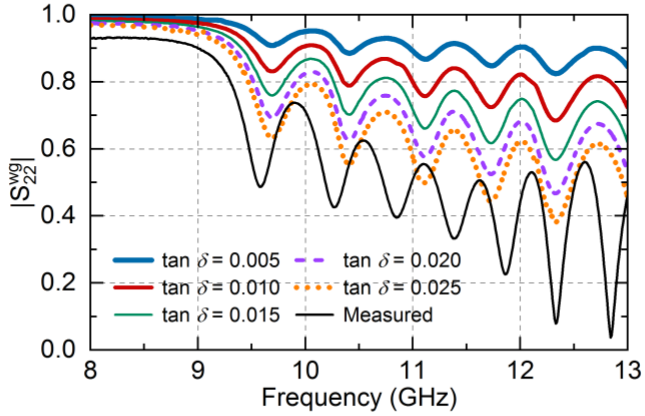

Fig. S2. Simulated values of the reflection coefficient of a WR-90 waveguide loaded with our designed LCBG for different values of dielectric loss tangent. From lowest to higher, they are shown as thick blue and red solid lines, and thin green, dashed purple, and dotted orange lines. Measured values are shown with solid black lines for reference. The influence of the losses in the reflectivity is clear. Both its average value and the depth of the valleys are severely affected as $\tan\delta$ increases, suggesting it is indeed the main limiting factor for *G*.

Table S1. BTAI *G* and *SNR* for different $\tan\delta$ values

$\tan\delta$	Parameter	Case 1	Case 2	Case 3	Case 4
0.005	G (dB)	6.53	5.04	8.16	7.07
	SNR (dB)	12.37	5.04	12.35	9.87
0.010	G (dB)	6.08	4.41	7.65	6.44
	SNR (dB)	11.78	4.42	11.87	9.47
0.015	G (dB)	5.68	3.82	7.11	5.86
	SNR (dB)	11.24	3.82	11.25	9.06
0.020	G (dB)	5.13	3.22	6.57	5.12
	SNR (dB)	10.60	3.22	10.75	8.72
0.025	G (dB)	4.51	2.62	5.96	4.31
	SNR (dB)	10.01	2.62	10.22	8.27
Measured	G (dB)	3.45	1.68	4.03	3.60
	SNR (dB)	8.89	12.34	7.10	12.12
